# Land use differentially affects fungal communities and network complexity in northeast China

**DOI:** 10.3389/fmicb.2022.1064363

**Published:** 2022-11-18

**Authors:** Yanxia Xu, Zhao Yang, Xiaolong Wang, Hua Chai, Shasha Li, Yue Wu, Ruoding Wang

**Affiliations:** Branch of Animal Husbandry and Veterinary of Heilongjiang Academy of Agricultural Sciences, Qiqihar, China

**Keywords:** land use, continuous cropping and alfalfa, grass, maize, fungal network

## Abstract

**Background:**

The soil fungal community is one of the most important drivers of the soil nutrient cycling that sustains plant growth. However, little research has been done on the effects of different land uses on soil fungal communities in northeast China.

**Methods:**

In this study, we conducted a field experiment to investigate the effects of continuous cropping of grass, maize, and alfalfa on their respective fungal communities and co-occurrence networks.

**Results:**

We showed that the physicochemical properties of the soil, such as nitrate (NO${}_{3}^{-}$N), available phosphorus, and soil pH, were the most important driving factors affecting the structure of the soil fungal community in different cropping systems. In addition, compared to the cultivation of grass and maize, the continuous cropping of alfalfa increased the abundance of several beneficial as well as pathogenic species, such as Mortierella and Gaiellales. In addition, the networks differed among plant species and according to the number of years of continuous cultivation.

**Conclusion:**

This suggests that the continuous cropping of alfalfa results in greater cooperation among fungi, which may be beneficial to the soil as well as to the development of the alfalfa.

## Introduction

Alfalfa (*Medicago sativa L*.) is a leguminous, perennial plant of great importance for livestock and agriculture and is therefore widely grown in many countries and regions (Han et al., 2005; Raiesi, 2007). The arid regions of northeast China are the main areas where alfalfa is grown. Due to the climatic specificity of the long winters in northeast China, livestock in the region rely heavily on summer pasture storage for forage (Chen et al., 2013). Alfalfa has a high yield and a comprehensive range of nutrients and can therefore reduce forage shortages for herbivores in winter (Su, 2007; Chen et al., 2013). As a result, perennial alfalfa is grown year after year in the region to meet the winter demand for fodder and to increase livestock productivity (Dong et al., 2003). However, this type of agricultural intensification has led to a loss of biodiversity (Sala et al., 2000; Romdhane et al., 2022). Moreover, the number of pathogenic microorganisms increases with the continuous planting of alfalfa, eventually leading to a decrease in yield, a phenomenon that is closely linked to soil microorganisms (Yan et al., 2012; Yao et al., 2019; Liu et al., 2020).

The number of years that alfalfa is grown is related to its productivity. Generally, alfalfa yields increase with the number of years planted; however, yields begin to decline when they reach a certain critical year, usually considered to be the 9^th^ year (Jiang et al., 2007; Li and Huang, 2008). In addition, continuous alfalfa cultivation significantly alters the physicochemical properties of the soil, which is significantly associated with yields (Ren et al., 2011). Previous studies have shown that planting alfalfa increases the organic matter and nitrogen content of the soil compared to virgin sandy soils. In addition, soil nutrients such as organic matter, nitrogen, and phosphorus increase with the number of continuous planting years. However, previous research has shown that soil nutrients tend to decrease after 10 continuous years of alfalfa cultivation (Jiang et al., 2007; Dong et al., 2016; Luo et al., 2018).

Soil microorganisms serve a crucial function in the maintenance of plant health by interacting with plants, participating in nutrient uptake and resisting stress, and responding rapidly to changes in the physical and chemical characteristics of the soil (Song et al., 2021). Differences in tillage systems, soil types, crop species, and cropping systems greatly influence the structure of the soil's microbial community (Zhou et al., 2018; Yao et al., 2019; Yuan et al., 2021). For example, one study observed that soil microbial biomass declined in the short term, but ultimately increased over the long term, in a context of continuous alfalfa cultivation (Jiang et al., 2007). Another study reported that continuous cultivation of alfalfa changed the microbial diversity by altering the physicochemical properties of the soil (Luo et al., 2018). Some studies have shown that continuous alfalfa cultivation can increase the relative abundance of *Paecilomyces phaeomycocentrospora* and *Fusarium* sp. and decrease the relative abundance of *Penicillium* sp. (Xu et al., 1995; Yao et al., 2019). However, other studies have found no effect of continuous alfalfa cultivation on soil microbiota structure (Hu and Wang, 1996). These different results might be attributed to heterogeneity among the soil types, sample collection times, and tillage systems used in these studies. Therefore, there is a need for more in-depth studies to investigate the barriers to continuous alfalfa cultivation under intensive tillage patterns.

Co-occurrence network analysis is a useful tool for exploring microbial associations and obtaining key information on microbial co-abundance communities associated with soil functions (Banerjee et al., 2018; Fan et al., 2021). One recent study used symbiotic networks to demonstrate that members of network modules were significantly associated with the genes involved in nutrient cycling after long-term fertilization, and that the number of members (operational taxonomic units, OTUs) in each module, rather than overall microbial diversity, influenced soil function (Fan et al., 2021). This raises the question of whether different land use practices alter the topological structures of networks, and what potential effects an altered network structure may have on soil function.

Here, we examined the influence of continuously cultivating grass, maize, and alfalfa (for 6, 10, 14, 20, and 30 years) on soil microbes and soil characteristics. Because grass grows without human intervention, we hypothesized that alfalfa and maize would have higher microbial diversity than grass, and that continuous planting of alfalfa would increase the complexity of the co-occurrence. The aims of this study were to explore the various changes in the structures of soil fungal communities across different cropping systems, and to assess the association between the physical characteristics of soil and the characteristics of its fungal community.

## Materials and methods

### Experimental site and design

The experimental site was located in Qiqihar, Heilongjiang Province, China. The experiments were carried out on natural meadow (Me), maize (Ma), and alfalfa (AC) in consecutive cultivation periods of different lengths: 6, 10, 14, 20, and 30 years, labeled C6, C10, C14, C20, and C30, respectively. Each treatment area was approximately 900 m^2^. Each summer, a compound fertilizer (N 18%, P_2_O_5_ 18%, and K_2_O 20%) of 18 kg mu^−1^ was applied to each treatment area, and the alfalfa was cut to the surface around July of each year.

### Soil sampling and measurement of soil characteristics

In total, 42 soil samples were collected with the z-stamping method at the end of June 2019. The plant roots and stones were filtered out using a 2-mm sieve. Soil samples of about 2 g each were filled into centrifuge tubes and then stored at −80°C for the DNA extraction and follow-up process. The remaining soil was stored at 4°C for testing of its physical and chemical properties. The pH of the soil was measured using a pH meter in a soil–water suspension (1:5 w/v). The total soil carbon and nitrogen contents were determined using an elemental analyzer (Jones and Willett, 2006). Nitrate (NO${}_{3}^{-}$-N) and ammonium (NH${}_{4}^{+}$-N) were extracted using 2.0 M potassium chloride and determined in a continuous flow analysis system. Furthermore, 0.5 M H_2_SO_4_-HClO_4_ and NaHCO_3_ were used to extract available and total phosphorus, respectively. HNO_3_-HClO_4_-HF and CH_3_COONH_4_ were used to extract total and available soil potassium, respectively, and two forms of potassium were determined using inductively coupled plasma emission spectrometry (ICPS-7500) (Lu, 1999).

### DNA extraction and sequencing

The Fast DNA Spin Kit (MP Biomedicals, USA) was used to extract total soil DNA. Fungal genes were amplified using the primers of ITS1 and ITS2 (Shi et al., 2020). PCR was performed using a 25 ml PCR mixture containing 10 ng DNA template, 10 mM of each primer, and 22 ml Platinum PCR SuperMix. The PCR program was 94°C for 4 min; 94°C for 20 s, 56°C for 10 s, 72°C for 15 s for 28 cycles; and 75°C extension for 10 min (Liu et al., 2015). Sequencing was performed on the Illumina MiSeq platform at Majorbio BioPharm Technology. The raw sequencing data were deposited in the NCBI BioProject, under accession number PRJNA890435.

Raw sequence data were processed using QIIME, version 1.17 (http://qiime.org/). PCR primer sequences and low-quality reads (length < 200 bp and mean quality score < 30) were trimmed using preliminary analyses. Sequence chimeras were removed using the UCHIME algorithm (Edgar et al., 2011). The sequences were then classified as operational taxonomic units (OTUs) using CD-HIT, with 97% similarity. Moreover, the trimmed sequences were phylogenetically assigned based on sequence alignment using RDP taxonomy with the UNITE database, version 7 (Cole et al., 2009; Li and Godzik, 2015).

The Shannon and Chao1 indices were calculated in QIIME. In addition, the canonical correspondence analysis, principal coordinate analysis (PCoA), and adonis test were performed using the “vegan” package, version R4.2.3. Using GenStat 13, one-way ANOVA was performed to analyze the differences in physicochemical properties of the soil samples and the relative abundances of various fungal genera. Fungal co-occurrence network analysis was performed for the fields of Me, Ma, and AC and for the treatments of AC6, AC10–20, and AC30. The OTU data were analyzed statistically in R and visualized in Gephi using the “psych” package (Jiang et al., 2017). The correlation between any two OTUs had to have a *p* < 0.05, and a Spearman's correlation coefficient of 0.7 or greater (Shi et al., 2020).

## Results

### Physicochemical characteristics of the soil

Compared to Me and Ma, AC had higher levels of soil nitrate, total potassium, available potassium, and pH, and lower levels of total nitrogen, total phosphorus, available phosphorus, and ammonium. In terms of the continuously cropped alfalfa, the soil nitrate, total potassium, total carbon, and nitrogen increased along with the number of years of continuous cropping, while ammonium, total phosphorus, and available phosphorus showed the opposite trend.

### Soil fungal diversity changed with different treatments

**Figure 1:**
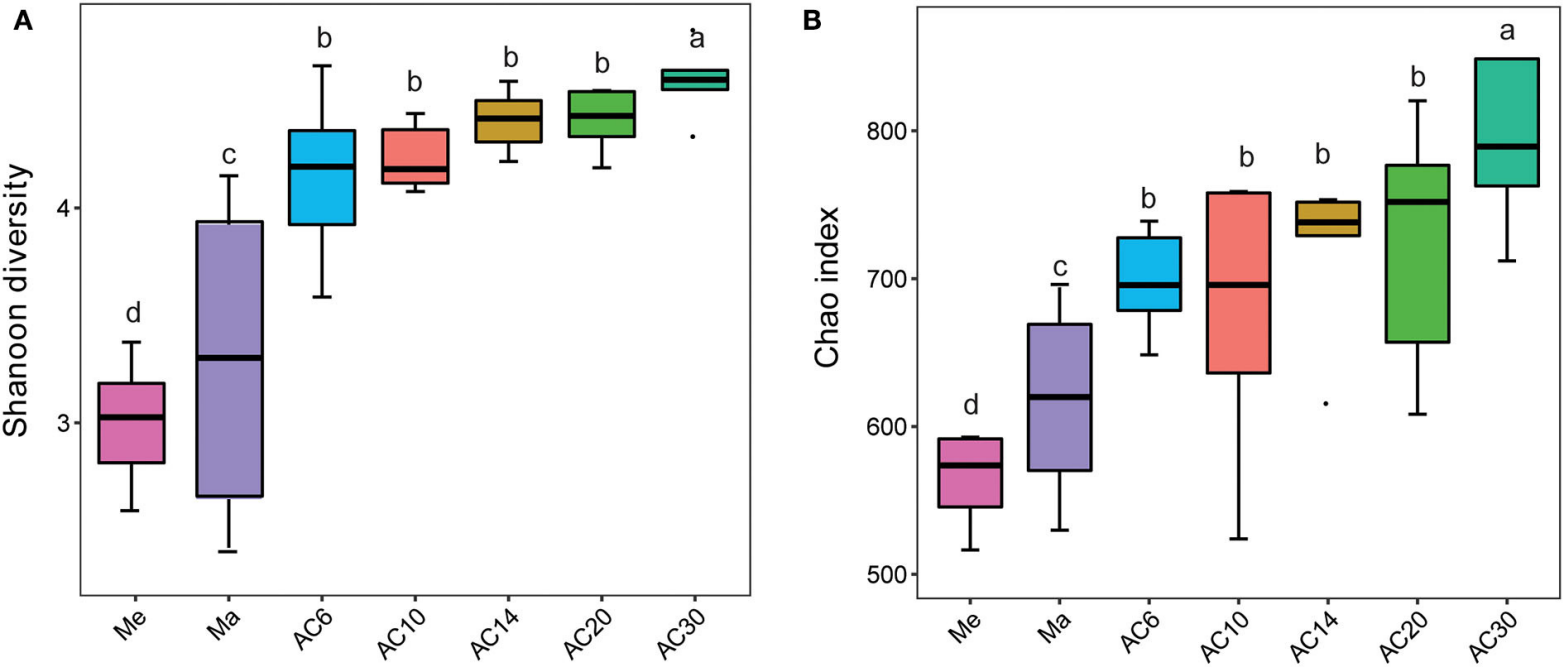
**(A)** Fungal Shannon diversity and **(B)** Chao index. The a–d letters indicate the significance of difference at *p* < 0.05 level.

**Figure 2:**
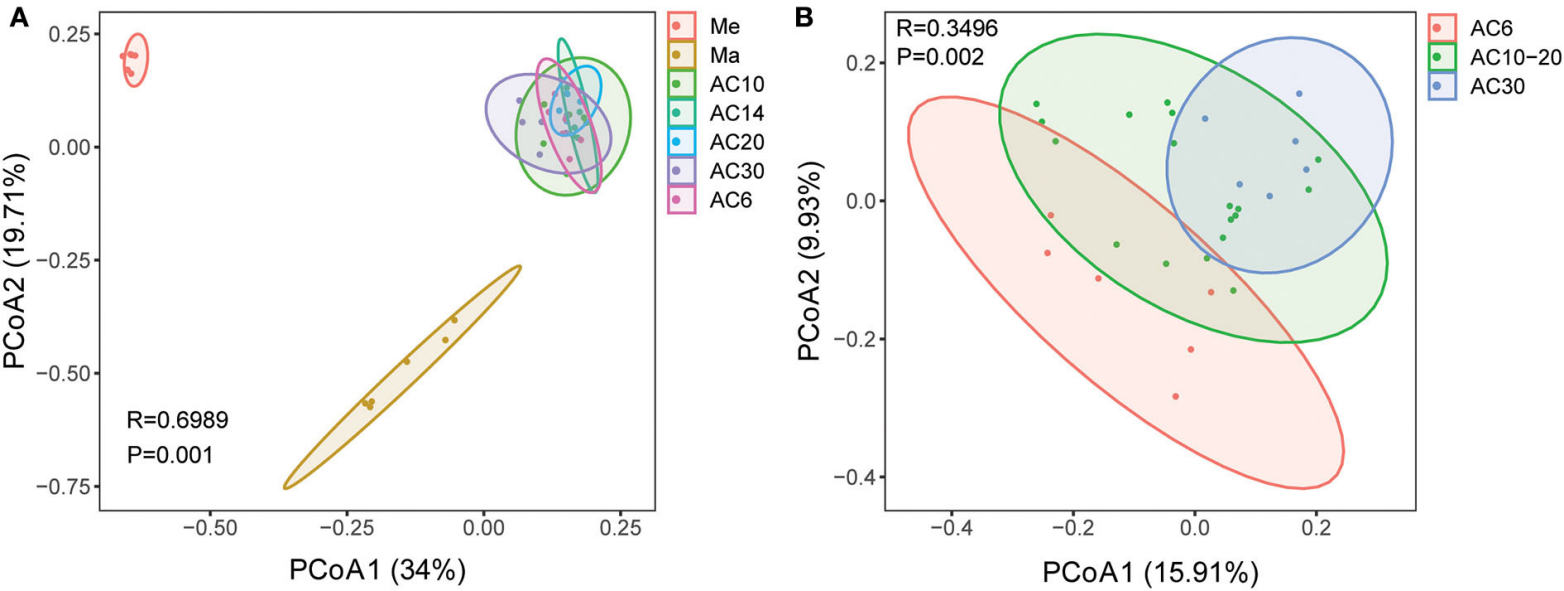
Principal coordinate analysis (PCoA) based on Bray–Curtis dissimilarities in the soil among different crop types **(A)** and different lengths of continuous cropping **(B)**.

Based on the results of the Shannon and Chao1 indices, the Me and AC30 treatments had the lowest and the highest fungal diversity, respectively (Figures 1A,B). Regarding the beta diversity, different cropping systems showed significantly different fungal community structures, according to the PCoA (PERMANOVA, *p* < 0.05) and adonis analysis (Figure 2A, Table 1). Moreover, the continuous cropping of alfalfa also had a significant influence on the fungal community structure (PERMANOVA, *p* < 0.05) (Figure 2B). Based on the PCoA results, all treatments were able to be divided into three distinct groups, i.e., Me, Ma, and AC6–30 (PERMANOVA, *p* < 0.05) (Figure 2A). We further performed PCoA on the samples from the continuous alfalfa planting and found that these samples could also be divided into three distinct groups, i.e., AC6, AC10–20 (i.e., 10, 14, and 20 years of continuous planting), and AC30, respectively (Figure 2B, Table 2). Moreover, using CCA analysis, we found that there was a significant association between the fungal community composition and soil characteristics (**Figure 6**). In detail, pH (r = 0.356; *p* = 0.03), total carbon (r = 0.553; *p* = 0.014), carbon:nitrogen ratio (r = 0.546; *p* = 0.03), nitrate (r = 0.691; *p* = 0.04), total potassium (r = 0.657; *p* = 0.03), available potassium (r = 0.564; *p* < 0.01), and available phosphorus (r = 0.543; *p* < 0.01) showed statistically significant associations with the fungal community composition.

**Table 1:** Effects of crop types, alfalfa continuous cropping times, and their interaction on the structure of soil fungal communities, based on adonis analysis.

**Factor**	**r**	***p***
Crop type (CT)	0.785	0.001^***^
Continuous years (CY)	0.624	0.02^*^
CT × CY	0.751	0.021^*^
**Pairwise comparison**		
Me vs. Ma	0.865	0.001^***^
Me vs. AC	0.763	0.001^***^
Ma vs. AC	0.567	0.003^**^
AC6 vs. AC10–20	0.529	0.02^*^
AC6 vs. AC30	0.688	0.001^***^
AC10–20 vs. AC30	0.587	0.002^**^

The

* ,

** , and

*** indicates the values of *p* < 0.05,

*p* < 0.01, and *p* < 0.001 respectively.

**Table 2:** Network topological characteristics of the different treatments.

**Network metrics**	**Me**	**Ma**	**AC**	**AC6**	**AC10–20**	**AC30**
Number of nodes	160	150	191	142	182	199
Number of edges	273	304	453	219	270	258
Number of positive correlations	243	291	369	208	246	230
Number of negative correlations	30	50	84	11	24	28
Average degree (avgK)	3.413	4.053	4.743	3.085	2.967	2.593
Average weighted degree	3.32	5.343	3.326	3.544	2.711	2.06
Network diameter	4	3	11	3	11	1
Graph density	0.021	0.027	0.025	0.022	0.016	0.013
Modularity (M)	0.967	1.256	1.371	1.033	1.16	3.556
Interconnecting piece	95	78	62	95	117	153
Average clustering coefficient (avgCC)	0.9	0.919	0.574	0.902	0.701	0.883
Average path length (APL)	1.291	1.222	4.255	1.215	4.336	1

### Specific fungal taxa changed with different treatments

**Figure 3:**
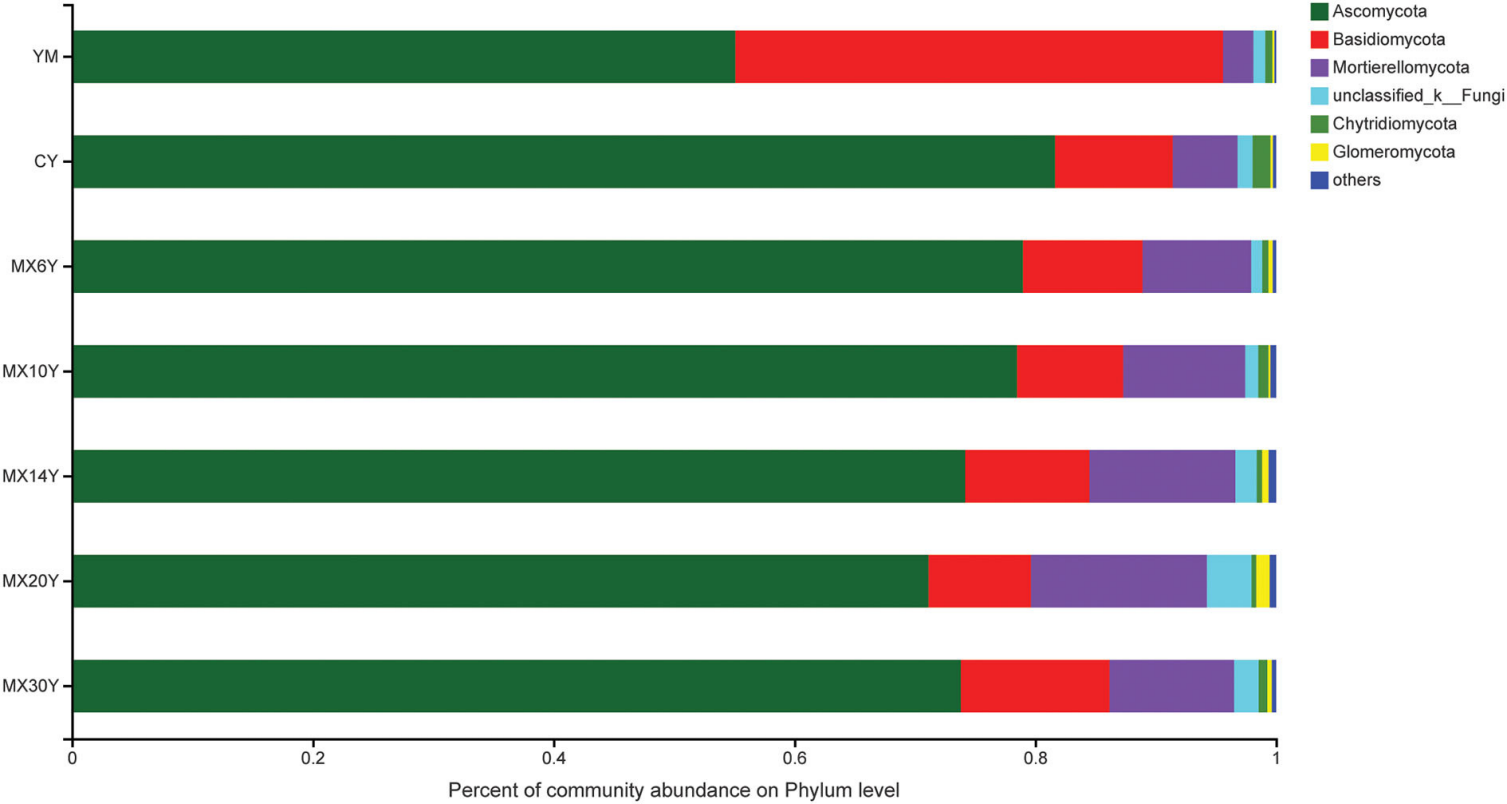
Composition of the fungal phylum level.

**Figure 4:**
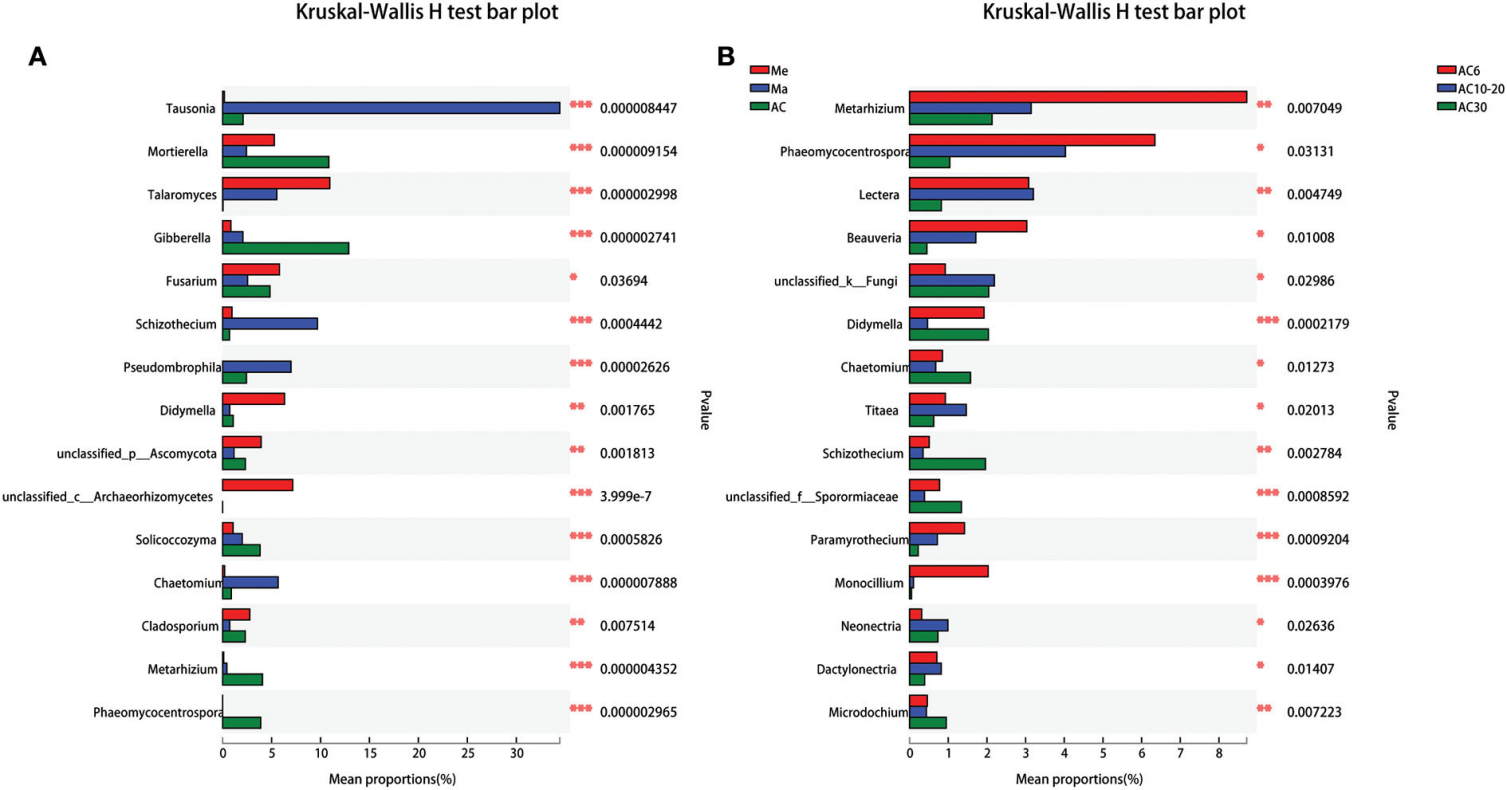
Effect of crop types **(A)** and continuous cropping years **(B)** on fungal genera.

Across all the treatments, *Ascomycota, Basidiomycota*, and *Mortierellomycota* were the dominant phyla, accounting for 93.45–96.32% of the whole community (Figure 3). In general, the relative abundance of *Basidiomycota* was much higher in the Me field compared with that in the Ma and AC fields, while the abundance of *Ascomycota* and *Mortierellomycota* was much higher in the AC treatments than in the Me and Ma fields. We then used the Kruskal–Wallis H-test to analyze different taxa on the genus level. Some genera, such as *Tausonia, Mortierella, Talaromyces, Gibberella, Fusarium*, and *Schizothecium*, showed significant difference among the various treatments (*p* < 0.05). In addition, other genera, such as *Metarhizium, Phaeomycocentrospora, Lectera, Beauveria, Didymella*, and *Schizothecium*, showed significant differences according to the number of years of continuous alfalfa cropping (Figure 4). Furthermore, the AC fields had a significantly higher relative abundance of *Mortierella, Gibberella, Solicoccozyma, Metarhizium*, and *Phaeomycocentrospora* compared with the other two fields and a lower relative abundance of *Tausonia, Talaromyces, Fusarium, Schizothecium, and Pseudobrophila* (Figure 4A). The relative abundance of *Metarhizium, Phaeomycocentrospora, Beauveria*, and *Monocillium* decreased with the number of years of continuous alfalfa cropping, while *Chaetomium, Titeae, Schizothecium*, and *Microdochium* showed the opposite trend (Figure 4B).

### Co-occurrence network in different treatments

**Figure 5:**
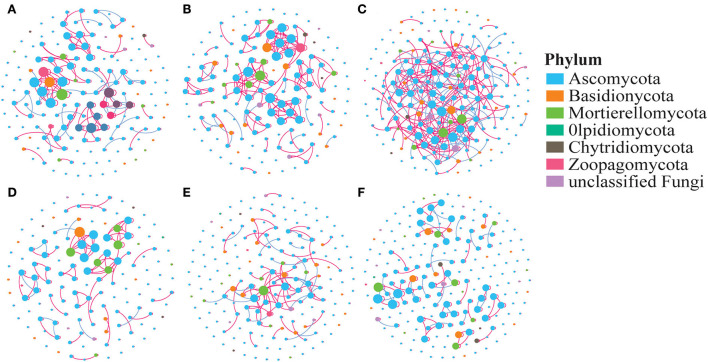
Co-occurrence network of the soil fungal community. **(A)** Me, **(B)** Ma, **(C)** AC, **(D)** AC6, **(E)** AC10-20, and **(F)** AC30. The nodes represent OTUs, and the edges represent the significant association between each OTU. The connection indicates a strong (Spearman's r > 0.7 or r < −0.7) and significant (*p* < 0.05) correlation. Red edges: positive connections; green edges: negative connections.

The co-occurrence network displays the relationship between the fungi in various treatments based on the OTU level (Figure 5). Neither the average degree (avgK) nor the clustering coefficient (avgCC) showed a significant difference among the treatments. The network modularity and the number of negative correlations were ranked as AC > Ma > Me. In a comparison of the AC6, AC10–20, and AC30 treatments, the negative correlations, modularity, and avgCC increased with the number of years of continuous cropping.

## Discussion

In the present study, fungal diversity was higher in the AC treatment than in the Me or Ma treatments, and the fungal diversity of alfalfa soils increased with subsequent years of planting. This result is in line with our first hypothesis, which suggests that alfalfa has more microbial species than grass and maize, and that continuous alfalfa planting is more beneficial to fungal diversity maintenance and soil sustainability, at least in terms of fungal diversity. Previous studies have found that continuously planted soybean has less soil microbial diversity than corn–soybean rotation systems (Liu et al., 2020). The number of years of continuous planting is also related to the microbial diversity of the soil (Liu et al., 2020). However, other studies have found no difference in microbial diversity between soils farmed continuously with soybean and soils farmed continuously with soybean–corn rotations (Li et al., 2010). These inconsistent results might be based on the soil types and the number of years of repeated harvests. Alterations in plant genotype may also be responsible for this result, given that microbial diversity has been shown to exhibit diverging trends in the context of successive cultivation of resistant and vulnerable cultivars (Yuan et al., 2021). Organic acids, phenols, and other compounds found in plant root exudates have an influence on microbial diversity in a range of agricultural situations (Tan et al., 2017; Lian et al., 2019; Liu et al., 2020; Shi et al., 2020). Furthermore, soil pH influences other soil characteristics that can directly or indirectly affect microbial diversity (Lian et al., 2019).

Regarding the beta diversity, the results from the principal coordinate analysis showed that crop types and continuous tillage time were the two most important factors affecting the structure of the soil fungal community (*p* < 0.05). Every species of plant releases a specific set of metabolites during growth, and this in turn allows its root system to provide a unique habitat for, and host different types of, soil fungal microorganisms. These microbes may also help plants absorb nutrients and resist stresses (Lian et al., 2019).

Moreover, our results were also consistent with previous studies that found that continuous crop planting also affects soil fungal community structure (Zhu et al., 2017; Yao et al., 2019; Yuan et al., 2021). This is mainly due to the effect of root exudates on soil microorganisms. For example, long-term continuous cultivation of soybean can lead to the accumulation of organic acids in the soil and ultimately cause soil acidification. This provides an ideal environment for pathogenic fungi to survive and alters the community structure of soil fungi, ultimately leading to reduced soil quality and crop yields (Zhu et al., 2017; Lian et al., 2019; Yuan et al., 2021).

**Figure 6:**
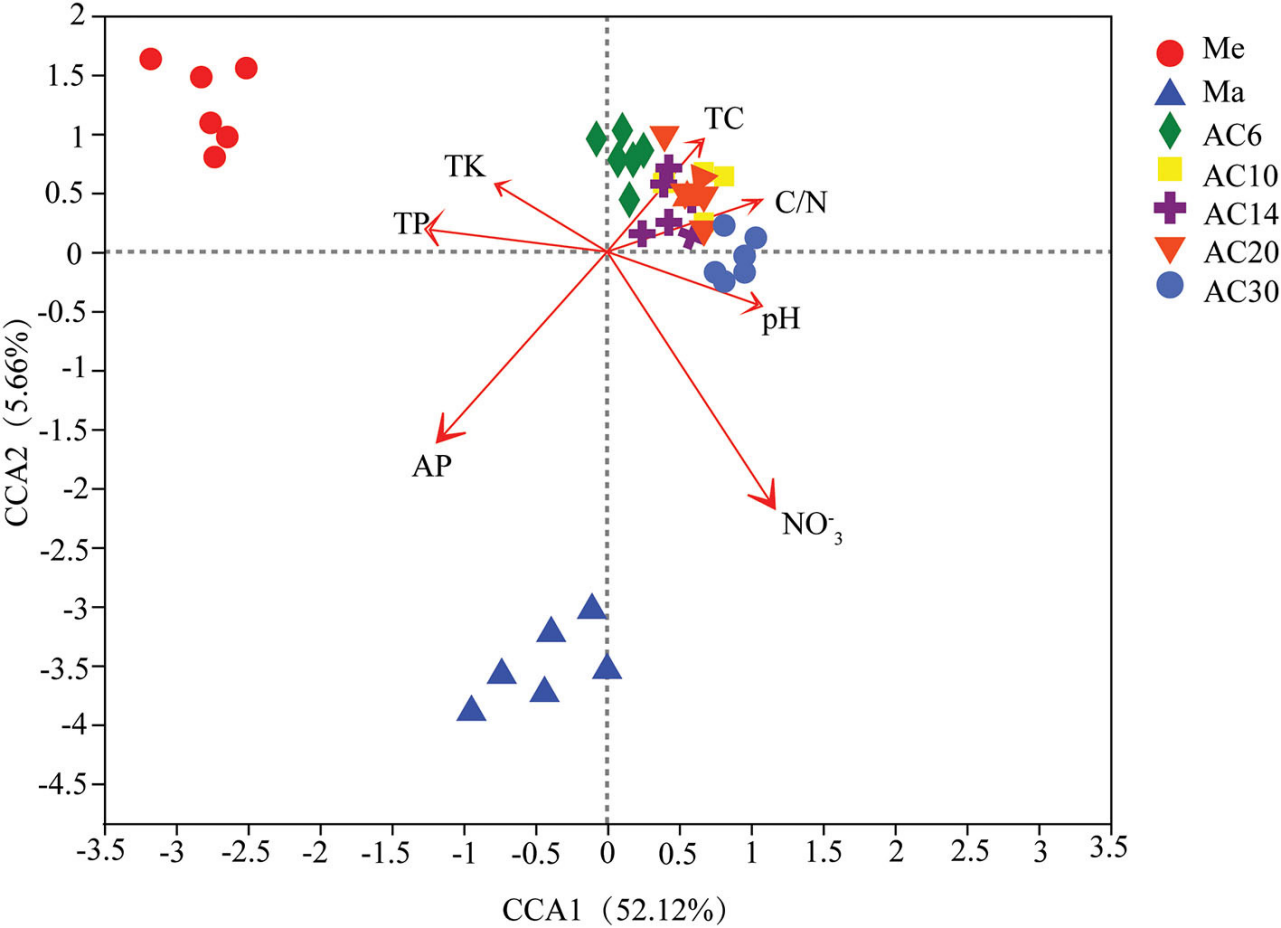
Relationship between soil properties and fungal community structure based on canonical correspondence analysis (CCA).

Furthermore, according to the CCA results, the physicochemical characteristics of our soil samples, such as NO${}_{3}^{-}$N, available phosphorus, and soil pH, were the main factors that altered the structure of the soil fungal community in the different treatments (Figure 6). This is similar to the findings of many previous studies, in which changes in tillage practices were shown to affect the microbial environment by altering the soil characteristics (Lian et al., 2019; Yao et al., 2019). Over all, our results also suggest that some important soil parameters changed significantly through continuous cropping over time, leading to changes in the fungal community. However, this change was not unidirectional, and longer periods of continuous cropping will perhaps lead to more positive developments for the soil microorganisms, such as increased diversity and significant enrichment of the beneficial fungi.

In AC soils, the relative abundance of *Ascomycota* was substantially higher compared to that of soils from the Ma and Mc systems (Figure 4). Many ascomycetes are plant-pathogenic, such as rice blast, black knot, the ergot fungi, and the powdery mildews, which suggests that continuous planting of alfalfa may have increased the abundance of potential pathogens, and influence the growth of alfalfa (Yuan et al., 2021). The relative abundance of *Mortierella, Gibberella, Solicoccozyma, Metarhizium*, and *Phaeomycocentrospora* was increased in AC fields compared to Me and Ma fields. It has been reported that *Mortierella* can survive under very unfavorable environmental conditions and make efficient use of carbon sources contained in polymers such as cellulose, hemicellulose, and chitin, and that it can synthesize phytohormones and 1-aminocyclopropane-1-carboxylic acid deaminase through improved access to bioavailable forms of phosphorus and iron in the soil, thereby protecting agricultural plants from pathogens (Ozimek and Hanaka, 2020). However, some pathogenic microbial species, such as *Gaiellales*, with high relative abundance in the AC treatment, can cause sear rot, suggesting that these fungi may suppress soil diseases, suggesting that these fungi may suppress soil diseases (Gómez Expósito et al., 2015). Therefore, it is likely that the changes in these fungi caused by the different treatments are related to the soil nutrient structure and the antagonistic activity of the plant pathogens.

In the present study, co-occurrence networks have helped us explore the complex relationships between fungi in different treatments in greater depth (Xue et al., 2018; Xiong et al., 2021). Our results show that both negative network correlations and modularity were significantly higher for AC than for Ma and Mc, which is consistent with our second hypothesis. This suggests that successive plantings of alfalfa promoted cooperation between fungi, which may be beneficial for alfalfa survival (Yao et al., 2019; Liu et al., 2020). Moreover, the difference in network topology of these treatmentsmay be due to the fact that certain microbial species are enriched to help the host increase nutrient uptake or resist stress, causing the structure of the microbial community to deviate from its original equilibrium (Lian et al., 2019). However, the results for the fungal networks alone are one-sided, as functional bacteria are also present in the soil. Therefore, in future studies, combined bacterial and fungal network analysis may yield more comprehensive results and a fuller assessment of the effects of different tillage practices on soil microbes.

In conclusion, alfalfa crop cultivation increased the alpha-diversity of soil fungi compared to grass and maize cultivation, and alpha-diversity increased further in continuous cropping systems, which is of great interest to maintain soil microbial diversity. The physicochemical properties of the soil, such as NO${}_{3}^{-}$-N, soil Ph, and available phosphorus, were the most important driving factors affecting the soil fungal community structure across the different cropping systems. Compared to the cultivation of grass and maize, the continuous cropping of alfalfa increased the abundance of several beneficial as well as pathogenic fungal species, such as *Mortierella* and *Gaiellales*. In addition, the networks differed among plant species and also among different lengths of time of continuous alfalfa cultivation. This suggests that the continuous cropping of alfalfa results in greater cooperation among fungi, which may be beneficial to the soil as well as to the development of the alfalfa.

## Data availability statement

The data presented in the study are deposited in the NCBI repository, accession number PRJNA890435.

## Author contributions

YX and ZY conceived of the presented idea. YX wrote the manuscript. YX, XW, HC, YW, RW, and SL verified the analytical methods. ZY supervised the findings of this work. All authors discussed the results and contributed to the final manuscript.

## Funding

This work was supported by the Heilongjiang Provincial Scientific Research Institute Scientific Research Operating Expenses Project (CZKYF2021-2-B025), Outstanding Youth Fund of Heilongjiang Academy of Agricultural Sciences (2020JCQN003), Grass-field Rotation Scientist Studio of Heilongjiang Province (202004), Heilongjiang Province Modern Agricultural Industry Technology Collaborative Innovation Promotion System Construction Project (Heilongjiang Agricultural Department Letter (2021) No. 1492), and Natural Science Foundation of Heilongjiang (YQ2022C033).

## Conflict of interest

The authors declare that the research was conducted in the absence of any commercial or financial relationships that could be construed as a potential conflict of interest.

## Publisher's note

All claims expressed in this article are solely those of the authors and do not necessarily represent those of their affiliated organizations, or those of the publisher, the editors and the reviewers. Any product that may be evaluated in this article, or claim that may be made by its manufacturer, is not guaranteed or endorsed by the publisher.
